# Physiological responses of *Lepidium meyenii* plants to ultraviolet-B radiation challenge

**DOI:** 10.1186/s12870-019-1755-5

**Published:** 2019-05-07

**Authors:** Thais Huarancca Reyes, Andrea Scartazza, Antonio Pompeiano, Lorenzo Guglielminetti

**Affiliations:** 1 Department of Agriculture, Food and Environment, University of Pisa, Via del Borghetto 80, 56124 Pisa, Italy; 2 Institute of Research on Terrestrial Ecosystems, National Research Council, Via Moruzzi 1, 56124 Pisa, Italy; 3 Center for Translational Medicine (CTM), International Clinical Research Center (ICRC), St. Anne’s University Hospital, 62500 Brno, Czech Republic; 4 Interdepartmental Research Center “Nutraceuticals and Food for Health”, University of Pisa, Via del Borghetto 80, 56124 Pisa, Italy

**Keywords:** Chlorophyll fluorescence, Gas exchanges, Maca, Multiple factorial analyses, Stress, Ultraviolet-B

## Abstract

**Background:**

Ultraviolet-B (UV-B) radiation can affect several aspects ranging from plant growth to metabolic regulation. Maca is a Brassicaceae crop native to the Andes growing in above 3500 m of altitude. Although maca has been the focus mainly due to its nutraceutical properties, it remains unknown how maca plants tolerate to harsh environments, such as strong UV-B. Here, we present the first study that reports the physiological responses of maca plants to counteract and recover to repeated acute UV-B irradiation.

**Results:**

In detail, plants were daily exposed to acute UV-B irradiation followed by a recovery period under controlled conditions. The results showed that repeated acute UV-B exposures reduced biomass and photosynthetic parameters, with gradual senescence induction in exposed leaves, reduction of young leaves expansion and root growth inhibition. Negative correlation between increased UV-B and recovery was observed, with marked production of new biomass in plants treated one week or more.

**Conclusions:**

A differential UV-B response was observed: stress response was mainly controlled by a coordinated source-sink carbon allocation, while acclimation process may require UV-B-specific systemic defense response reflected on the phenotypic plasticity of maca plants. Moreover, these differential UV-B responses were also suggested by multifactorial analysis based on biometric and physiological data.

**Electronic supplementary material:**

The online version of this article (10.1186/s12870-019-1755-5) contains supplementary material, which is available to authorized users.

## Background

Maca (*Lepidium meyenii* Walpers or *Lepidium peruvianum* Chacón) is a Brassicaceae crop and herbaceous plant native to the central highlands of Peruvian Andes. The plant consists of frilly leaves forming a tight rosette, and an enlarged fleshy underground organ made up of the taproot and hypocotyl commonly referred to as ‘hypocotyl’ [18]. Since pre-Columbian times, maca hypocotyls have not been only used as food due to its nutrients content, but also as traditional medicine [6]. Several active components have been identified in the hypocotyls including glucosinolates, macamides, macaenes, sterols, phenolics, essential oils (mainly phenylacetonitrile) and polysaccharides, which are related to their medicinal properties [19]. Recently, maca has gained attention due to its potential health benefits which make it an attractive plant for the nutraceutical industry [3].

Maca is the only *Lepidium* species which shows robust resistance to extreme environmental stresses growing at elevations of 3500 to 4500 m above the sea level where only highland grasses and few hardy potatoes can survive. Its ecosystem is characterized by freezing temperatures, high humidity, strong wind, high ultraviolet (UV) radiation, and relative infertile and rocky soil [26]. UV-B radiation represents a small fraction of the solar spectrum; however, its high energy photons have a substantial impact on living organisms [22]. Exposure to UV-B has multiple effects on plants including photomorphogenesis and damaging responses mediated by UV-B-specific and nonspecific signaling pathways. Thus, low doses of UV-B mainly induce photomorphogenic changes via the UV-B RESISTANCE LOCUS 8 (UVR8) photoreceptor, while high doses cause damage to biomolecules by the production of reactive oxygen species (ROS) that activate additional stress signaling such as DNA damage signaling, defense and wound signaling, and hormone signaling pathways [8, 9, 13, 34]. However, these UV-B mediated responses are not mutually exclusive; even more tend to overlap depending on the threshold doses of species [33].

Although maca has been the focus of recent attention mainly due to several studies on its nutraceutical properties, it remains unknown how maca plants tolerate to harsh environments, such as strong UV-B radiation. Interestingly, a recent genomic study identified some duplicated genes with functions in morphological adaptation and abiotic response in maca plants under natural environment [40]. Therefore, the objective of the present study is to evaluate the physiological traits of this important crop in response to UV-B. To this aim, maca plants of the same age were daily exposed to acute UV-B irradiation (1, 3, 7, 10 or 14 days) followed by a recovery period of 2 weeks under controlled conditions. Comparative physiological responses, including biomass, carbohydrates, fluorescence parameters of photosystem II (PSII) and gas exchanges, were evaluated in both untreated and UV-B treated plants before and after recovery. Here, we presented the first study that reports the physiological responses of maca plants to counteract and recover to repeated acute UV-B irradiation.

## Results

### Biometric analysis

**Fig. 1:**
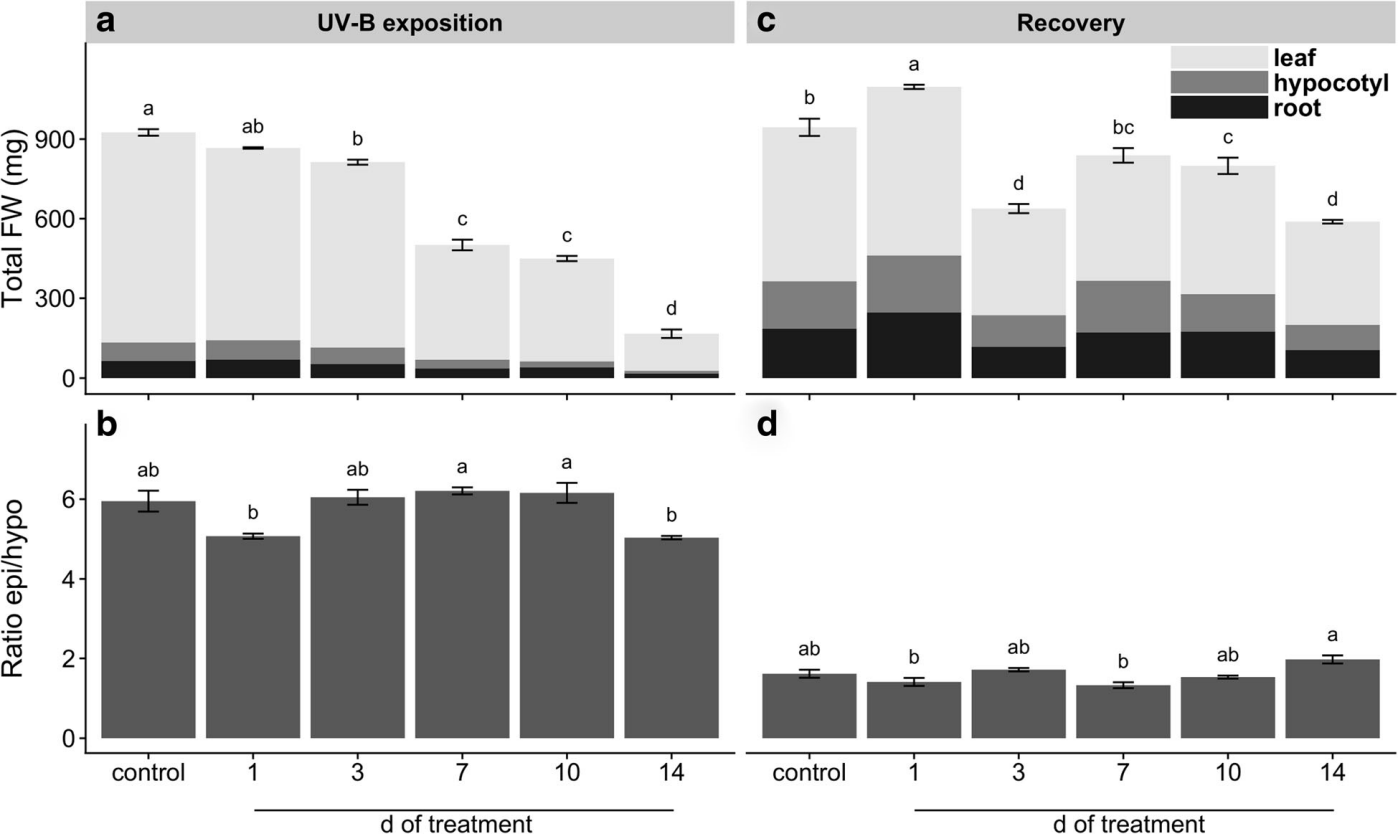
Effects of UV-B irradiation on biometric traits before and after recovery. Total FW (**a**, **c**) and ratio between FW of epigeal to hypogeal organs (**b**, **d**) of UV-B treated and control plants before (**a**, **b**) and after recovery (**c**, **d**). Treated plants were exposed to 100 μmol quanta m^− 2^ s^− 1^ and 6.08 kJ m^− 2^ d^− 1^ UV-B. UV-B was daily applied following a time course from 1 to 14 days. Control plants did not receive UV-B radiation. For recovery test, UV-B treated plants were transferred to control conditions for two weeks. Error bars represent the standard error of the mean (*n* = 3). The different letters indicate significant differences between means tested using Tukey’s HSD tests (*P* < 0.05). FW, fresh weight

Maca plants exposed to UV-B irradiation showed a clear reduction of total fresh weight (FW) in comparison to the control which was highly pronounced with the irradiation time (Fig. 1a). Evaluation of the ratio between the FW of epigeal with respect to hypogeal organs revealed that plants irradiated with UV-B for 1 and 14 d showed lower but not significant difference in comparison with that of the control and 3 d UV-B, while their ratio were significantly lower than 7 and 10 d UV-B exposed plants (Fig. 1b). UV-B treated plants after 2 weeks of recovery under control conditions showed different total FW pattern with respect to the results obtained just after treatment. UV-B irradiation for 1 d resulted in an increase of FW in comparison to the control plants, while other UV-B exposition periods resulted in a decrease of FW (Fig. 1c). Evaluation of the FW ratio between epigeal and hypogeal organs after 2 weeks of recovery did not show significant differences between UV-B treated and control plants (Fig. 1d).

### Total soluble sugars

**Fig. 2:**
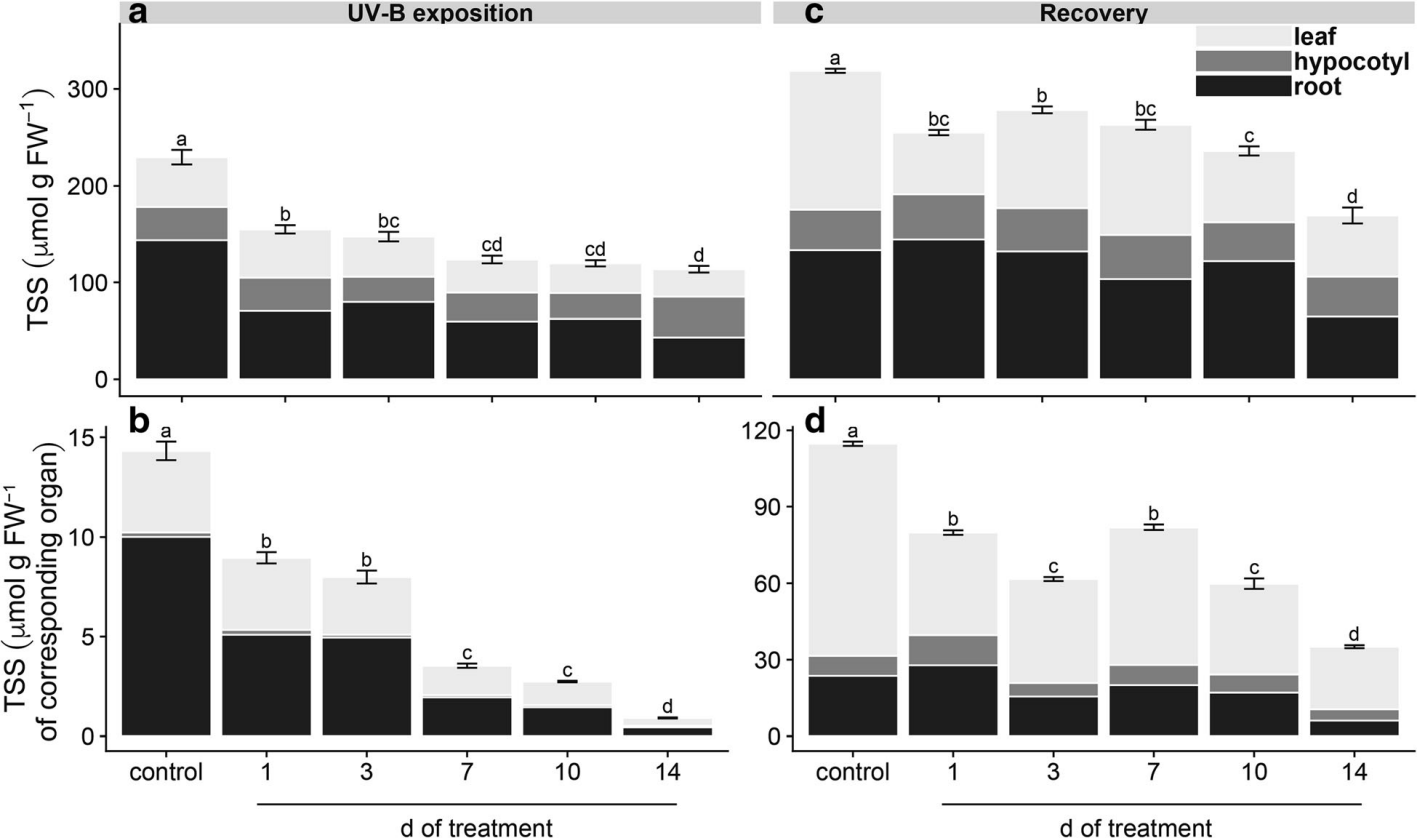
Effects of UV-B irradiation on total soluble sugars (TSS) before and after recovery. TSS content expressed in μmol g^− 1^ FW (**a**, **c**) and μmol g^− 1^ FW of corresponding organ (**b**, **d**) was determined in UV-B treated and control plants before (a, b) and after recovery (c, d). Treated plants were exposed to 100 μmol quanta m^− 2^ s^− 1^ and 6.08 kJ m^− 2^ d^− 1^ UV-B. UV-B was daily applied following a time course from 1 to 14 days. Control plants did not receive UV-B radiation. For recovery test, UV-B treated plants were transferred to control conditions for two weeks. Error bars represent the standard error of the mean (*n* = 3). The different letters indicate significant differences between means tested using Tukey’s HSD tests (*P* < 0.05). FW, fresh weight

UV-B irradiation in maca plants resulted in a significant and gradual decrease of total soluble sugars (TSS) content in comparison to that of the control when exposition time was prolonged (Fig. 2a). Similar pattern was observed when the content of TSS was expressed per FW of corresponding organ (Fig. 2b), which was tightly correlated with the steady reduction in FW of treated plants with respect to the control (Fig. 1a). Recovery test in UV-B treated plants showed a general increase of TSS with respect to plants just after treatment; however, these values were lower in comparison to the control, rising to the lowest level in 14 d treated plants (Fig. 2c). Similar results were obtained when TSS content was expressed per FW of corresponding organ with a general reduction during the UV-B exposition in comparison to the control (Fig. 2d). Moreover, the distribution of TSS between source and sink tissues after recovery were more evident when it was expressed per organ than FW (Fig. 2c, d).

### Starch content

**Fig. 3:**
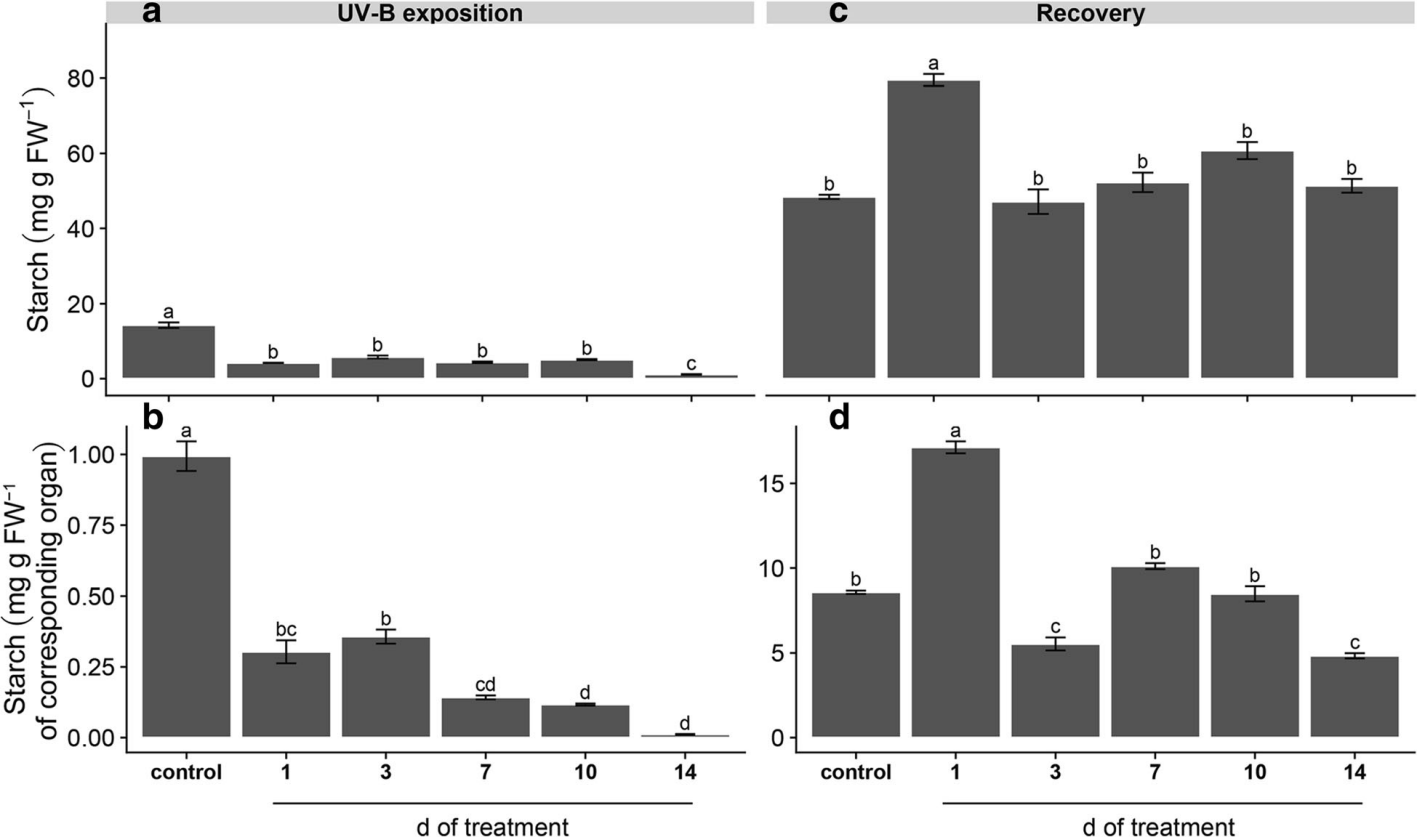
Effects of UV-B irradiation on starch content before and after recovery. Starch content expressed in mg g^− 1^ FW (**a**, **c**) and mg g^− 1^ FW of corresponding organ (**b**, **d**) was determined in hypocotyls of UV-B treated and control plants before (**a**, **b**) and after recovery (**c**, **d**). Treated plants were exposed to 100 μmol quanta m^− 2^ s^− 1^ and 6.08 kJ m^− 2^ d^− 1^ UV-B. UV-B was daily applied following a time course from 1 to 14 days. Control plants did not receive UV-B radiation. For recovery test, UV-B treated plants were transferred to control conditions for two weeks. Error bars represent the standard error of the mean (*n* = 3). The different letters indicate significant differences between means tested using Tukey’s HSD tests (*P* < 0.05). FW, fresh weight

Hypocotyls of control plants contained the highest amount of starch in comparison to treated plants, which showed approx. 30% of control level in plants exposed to 1, 3, 7 and 10 d UV-B and reached to the lowest starch level when UV-B exposition was extended to 14 d (Fig. 3a). A gradual reduction of starch content was observed in UV-B irradiated plants with respect to the control when starch was expressed per FW of organ (Fig. 3b). Results after recovery showed that only 1 d-treated plants contain higher starch level than the control, while the other UV-B treated plants showed similar starch level as the control (Fig. 3c). Evaluation of starch content after recovery expressed per FW of organ revealed that 1 d-treated plants contain the highest starch level (twice with respect to the control), while 3 and 14 d of treatment presented the lowest starch content (approx. 60% of control or 30% of 1 d-treated plants), and 7 and 10 d-treated plants contained similar starch level as the control (Fig. 3d).

### Chlorophyll *a* fluorescence

**Fig. 4:**
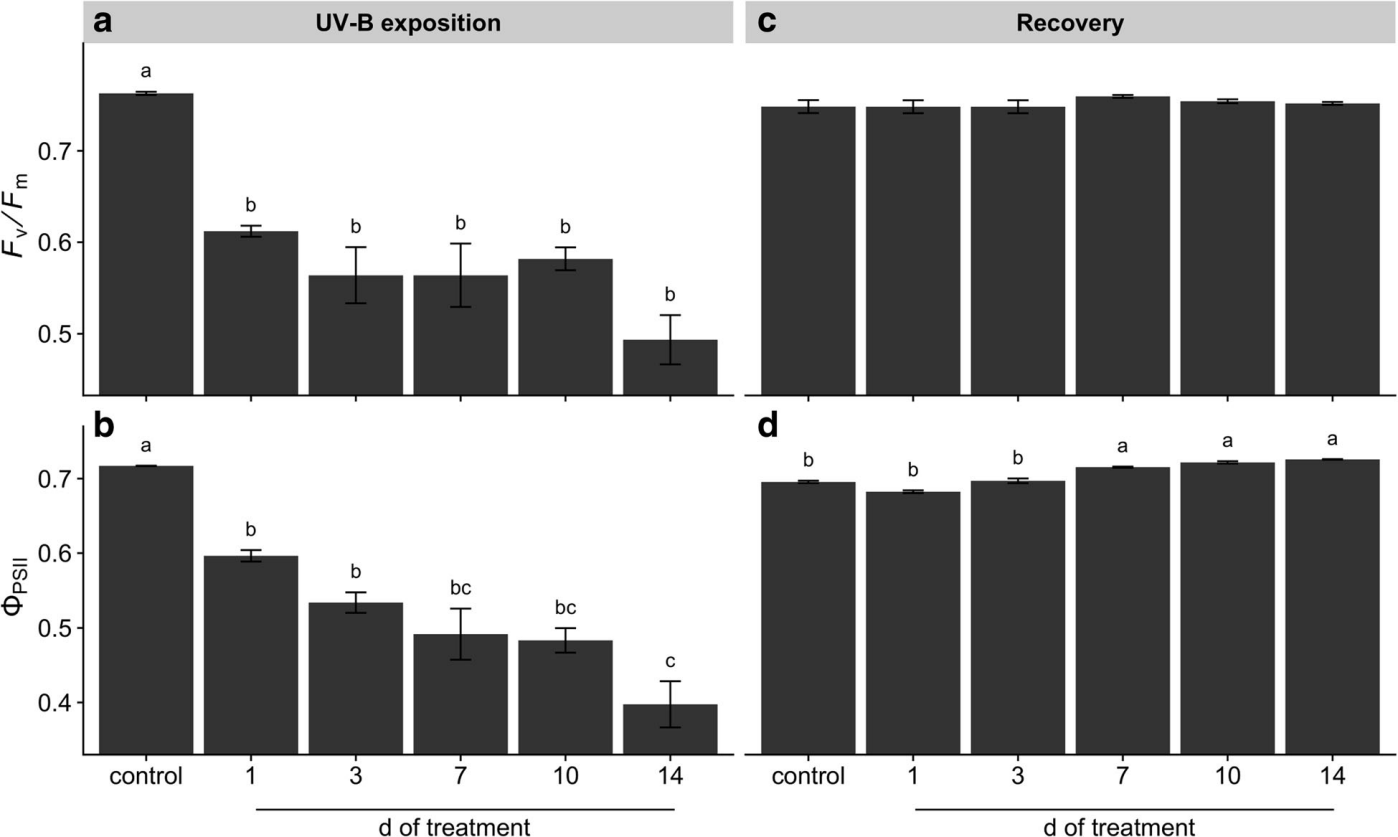
Effects of UV-B irradiation on the maximum (*F*_*v*_*/F*_*m*_) and actual (Φ_PSII_) efficiency of PSII photochemistry. *F*_*v*_*/F*_*m*_ (**a**, **c**) and Φ_PSII_ (**b**, **c**) were evaluated in leaves of UV-B treated and control plants before (**a**, **b**) and after recovery (**c**, **d**). Treated plants were exposed to 100 μmol quanta m^− 2^ s^− 1^ and 6.08 kJ m^− 2^ d^− 1^ UV-B. UV-B was daily applied following a time course from 1 to 14 days. Control plants did not receive UV-B radiation. For recovery test, UV-B treated plants were transferred to control conditions for two weeks. Error bars represent the standard error of the mean (*n* = 3). The different letters indicate significant differences between means tested using Tukey’s HSD tests (*P* < 0.05)

Evaluation of chlorophyll *a* fluorescence parameters showed that UV-B exposure strongly decreased the maximum PSII photochemical efficiency (*F*_*v*_/*F*_*m*_) with respect to the control with a clear negative effect on the actual photon yield of PSII photochemistry (Φ_PSII_) values which gradually decreased with the exposition time (Fig. 4a, b). Recovery of UV-B-treated plants showed that *F*_*v*_/*F*_*m*_ parameter was restored to optimal level without differences with respect to the control (Fig. 4c). However, Φ_PSII_ in 7, 10 and 14 d-UV-B treated plants after recovery showed the highest values, followed by control, 1 and 3 d-UV-B treated plants (Fig. 4d).

### Leaf gas exchange

**Fig. 5:**
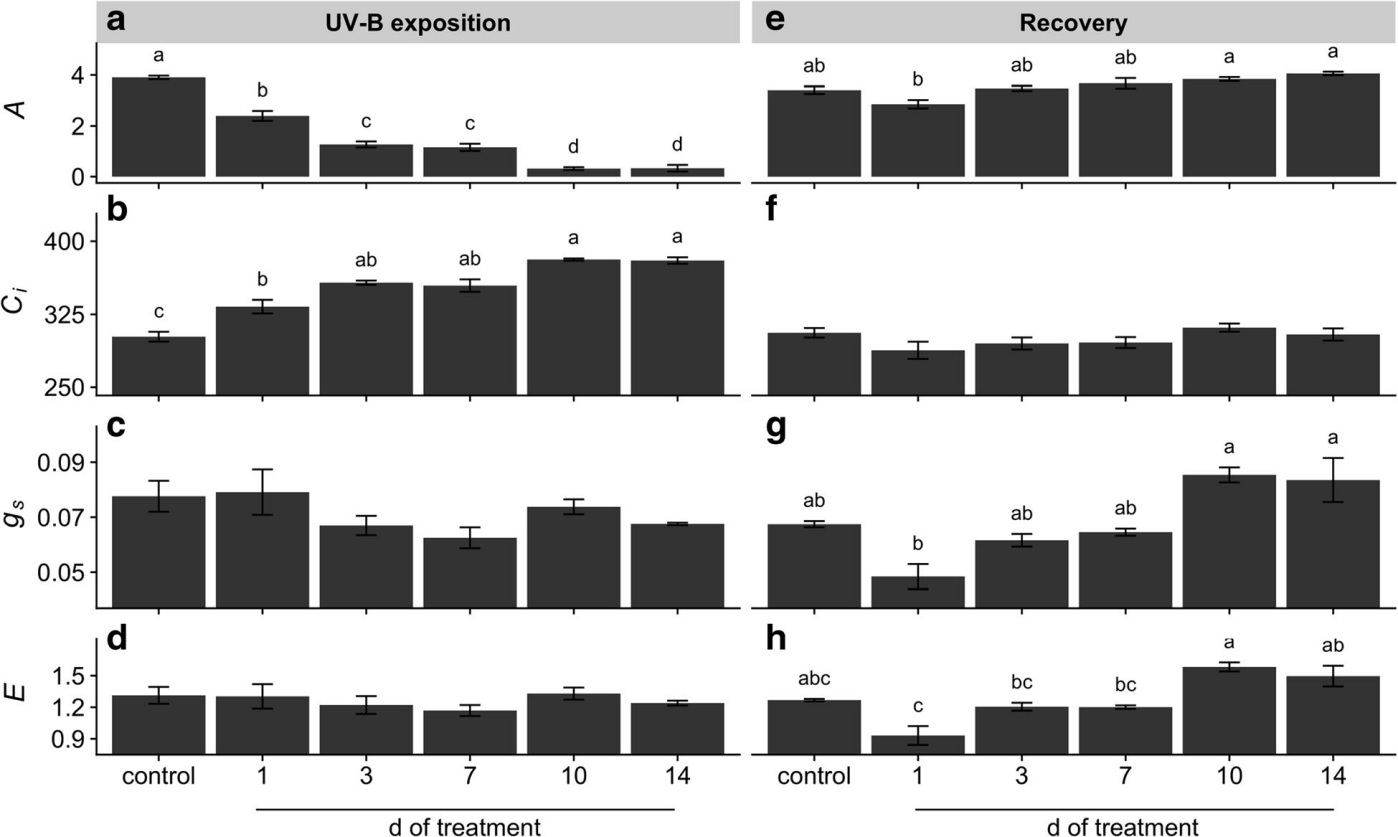
Effects of UV-B irradiation on maca leaf gas exchanges. Parameters of CO_2_ assimilation rate (*A*, μmol CO_2_ m^− 2^ s^− 1^) (**a**, **e**), intercellular CO_2_ concentration (*C*_*i*_, μmol CO_2_ mol^− 1^) (**b**, **f**), stomatal conductance (*g*_*s*_, mol H_2_O m^− 2^ s^− 1^) (**c**, **g**) and transpiration rate (*E*, mmol H_2_O m^− 2^ s^− 1^) (**d**, **h**) were evaluated in leaves of UV-B treated and control plants before (**a**-**d**) and after recovery (**e**-**h**). Treated plants were exposed to 100 μmol quanta m^− 2^ s^− 1^ and 6.08 kJ m^− 2^ d^− 1^ UV-B. UV-B was daily applied following a time course from 1 to 14 days. Control plants did not receive UV-B radiation. For recovery test, UV-B treated plants were transferred to control conditions for two weeks. Error bars represent the standard error of the mean (*n* = 3). The different letters indicate significant differences between means tested using Tukey’s HSD tests (*P* < 0.05)

**Fig. 6:**
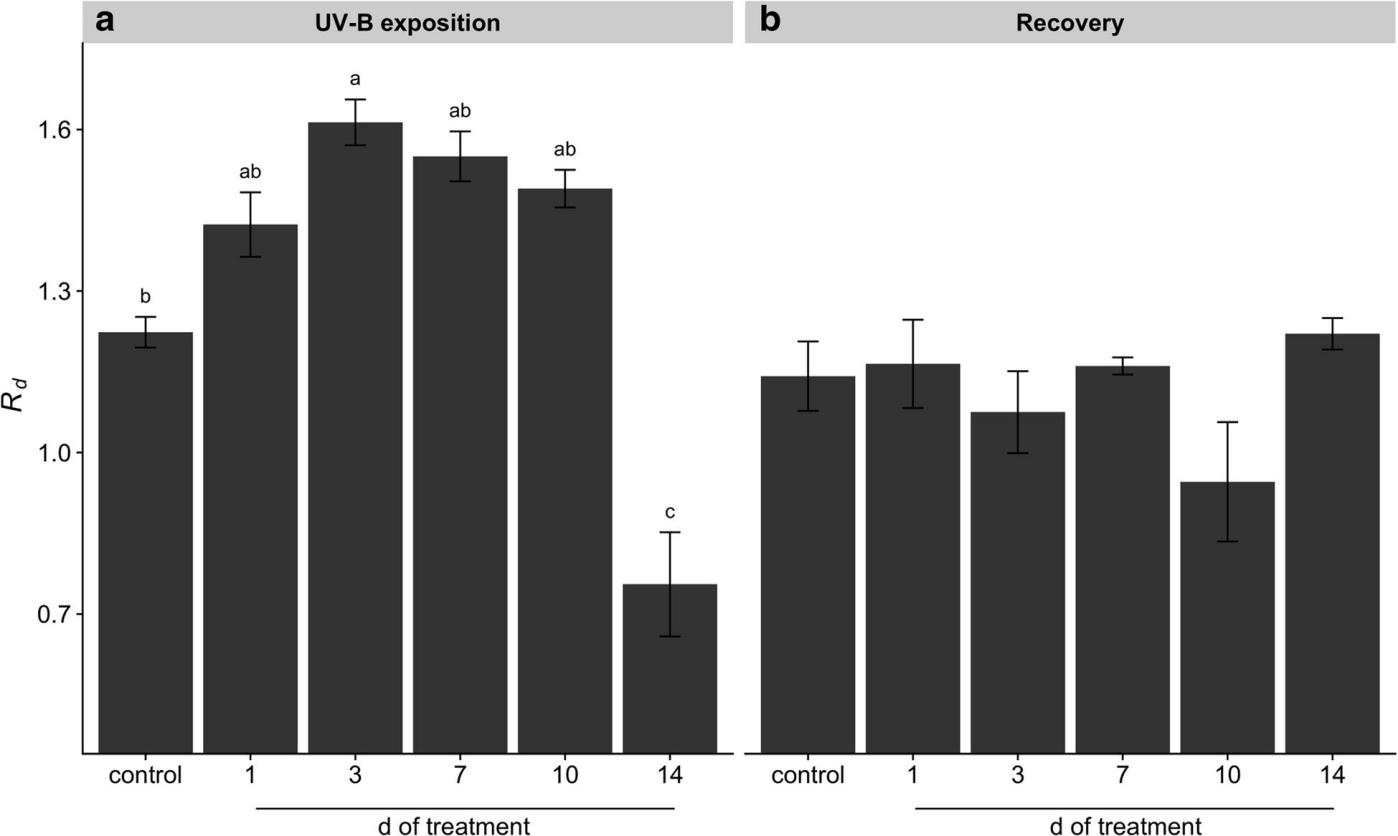
Effects of UV-B irradiation on dark respiration (*R*_*d*_) of maca. *R*_*d*_ (μmol CO_2_ m^− 2^ s^− 1^) was evaluated in leaves of UV-B treated and control plants before (**a**) and after recovery (**b**). Treated plants were exposed to 100 μmol quanta m^− 2^ s^− 1^ and 6.08 kJ m^− 2^ d^− 1^ UV-B. UV-B was daily applied following a time course from 1 to 14 days. Control plants did not receive UV-B radiation. For recovery test, UV-B treated plants were transferred to control conditions for two weeks. Error bars represent the standard error of the mean (*n* = 3). The different letters indicate significant differences between means tested using Tukey’s HSD tests (*P* < 0.05)

Gas exchange analysis showed a gradual decrease of CO_2_ assimilation rate (*A*) with the UV-B exposition time leading to significantly lower values than the control (Fig. 5a). Conversely, the evaluation of intercellular CO_2_ concentration (*C*_*i*_) resulted in a gradual increase with the UV-B treatment with the lowest values obtained in control plants (Fig. 5b). Analysis of stomatal conductance (*g*_*s*_) and transpiration rate (*E*) did not present significant differences between treatments and control condition (Fig. 5c, d). Evaluation of dark respiration (*R*_*d*_) in UV-B treated plants showed a transient increase reaching the highest value at 3 d, followed by a slight decrease at 7 and 10 d which were not significantly different than the control, and a subsequent sharp decrease at 14 d of treatment (Fig. 6a). Differently, recovery of 1 d UV-B treated plants showed lower but not significant different *A* than the control, while increasing UV-B exposition caused a gradual increase of *A* rising to significant different values with respect to recovered 1d-treated plants (Fig. 5e). Similar pattern to *A* was observed in the evaluation of *g*_*s*_ and *E* after recovery (Fig. 5g, h), while *C*_*i*_ and *R*_*d*_ values resulted in not statistical differences between treated and untreated plants after recovery (Fig. 5f, 6b).

### Multiple factorial analyses

**Fig. 7:**
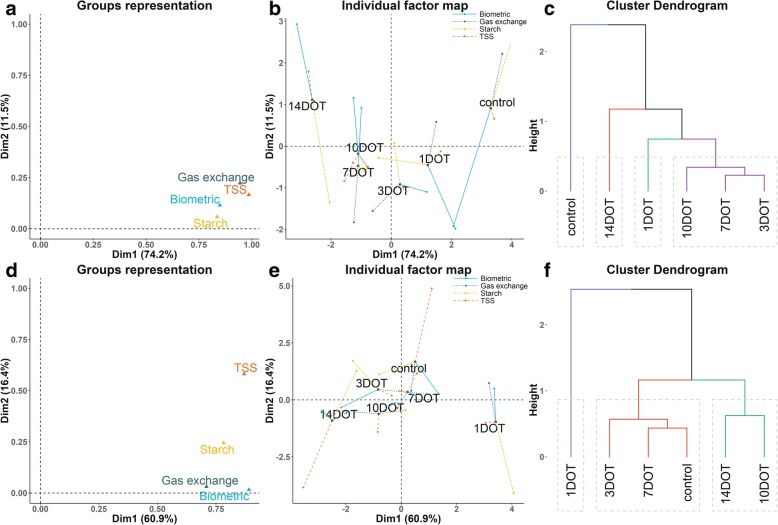
Multiple factor analysis (MFA) of biometric and physiological data in maca. Control and UV-B irradiated plants at the end of the treatment (upper panels) and after recovery (below panels). Representation of groups of variables (**a**, **d**). Key: blue “Biometric”, Leaf, root and hypocotyl FW; yellow “Starch”, starch expressed in mg g^− 1^ FW and mg g^− 1^ FW of corresponding organ content; orange “TSS”, total soluble sugars expressed in μmol g^− 1^ FW and μmol g^− 1^ FW of corresponding organ; grey “Gas exchange”, CO_2_ assimilation rate (*A*), stomatal conductance (*g*_*s*_), intercellular CO_2_ concentration (*C*_*i*_), transpiration rate (*E*), dark respiration (*R*_*d*_), maximum (*F*_*v*_*/F*_*m*_) and actual (Φ_PSII_) efficiency of PSII photochemistry. Score plot describing the exposure times and groups of variables of the two-first principal components (**b**, **e**). Hierarchical clustering of exposure times based on their biometric and physiological traits (**c**, **f**). DOT, days of UV-B treatment

Multiple factorial analyses (MFA) revealed the relationship between the exposure time fingerprints obtained from biometric and physiological data recorded after treatment and recovery. The coordinates of the four groups of variables were displayed and used to create a map of the groups (Groups representation; Fig. 7a, d). The coordinates were calculated using the first two dimensions of the MFA (Dim 1 and 2 on the diagram), which resumed 85.75 and 77.31% of the total variance of the dataset. As to the contribution of individual groups of variables after treatment, an overall equilibrium was recorded for axis 1 (Fig. 7a). Different conclusions can be drawn regarding the contribution of each group of variables to axis 2. The contribution of gas exchange appears to be the most significant (39.64%), compared to the low contribution of starch (10.27%), the latter the least useful groups of variables for discriminating among the analyzed exposure times on the axis 2 of the MFA.

These data can be read in the same way as data in a normal PCA: the individual trait classes correspond to the correlation coefficients between these variables and the exposure time fingerprints, meanwhile the length and the direction of the vectors are directly correlated to their significance within each exposure time. Factorial axis 1 (74.21% of the variance) clearly separated the individuals according to the exposure time, with the exception of 7 and 10 days of UV-B treatment (DOT) (Individual factor map; Fig. 7b). The hierarchical clustering provided by the MFA highlighted the overall performance of the treatments obtained through the single analysis of the biometric and physiological data (Hierarchical clustering; Fig. 7c). The three phylogenetic trees show that the control, 1 DOT, as well as 14 DOT are clearly differentiated from the other treatments analyzed, while the treatments from 3 to 10 DOT cannot be separated on the basis of their biometric and physiological traits.

On the other hand, MFA revealed marked changes after recovery. As to the contribution of individual groups of variables, a general equilibrium can be still observed for axis 1, although for axis 2, the contribution of TSS appears as to be the most statistically significant (66.59%) compared with starch (28.00%), and the other variables (2.70% on average) (Fig. 7d). Both axes contributed to separate the exposure times, except the 1 DOT that is clearly separated according to axis 1 (Fig. 7e). The hierarchical clustering provided by the MFA after recovery highlighted that 1 DOT clearly differentiated from the other exposure times analyzed, 14 DOT share more similarity to 10 DOT, while the control, 3 and 7 DOT clustered together on the basis of their biometric and physiological traits (Fig. 7f).

## Discussion

Since its initial reports in 1960s [18], maca has been the focus of recent attention mainly due to several studies on its nutraceutical properties [19]. However, no investigations were performed in order to understand the different strategies of maca plants to tolerate harsh environments, such as strong UV-B irradiance. To the best of our knowledge, this is the first study that reports the physiological responses of maca plants to counteract and recover to repeated acute UV-B irradiation. It should be mentioned that in this study, we performed the experiments under controlled conditions to exclude other additional stresses that are common in the natural environment where this species is cultivated. In addition, the unbalanced visible radiation/UV-B is an experimental strategy commonly used (i.e. [11, 35, 39]) to magnify the effect of a single stress in plants, which can be useful for elucidation of response mechanisms.

Our results showed that repeated acute UV-B exposures reduced plant biomass (Fig. 1) due to a gradual induction of leaf senescence, reduction of young leaves expansion and root growth inhibition (Additional file 1: Figure S1). Leaf senescence phenotype might be related to ROS-triggered programmed cell death (PCD) in well-expanded and UV-B-exposed leaves (Additional file 2: Figure S2). In fact, ROS have been suggested as key inducers of developmental and/or environmental PCD [23]. Moreover, a recent review pointed that higher natural and laboratory-applied UV-B doses can induce ROS production, damage to cell membranes, proteins and DNA through UV-B non-specific signaling pathways [2]. Thus, this ultimate PCD in response to repeated UV-B irradiation might be associated with the massive accumulation of ROS (Additional file 2: Figure S2). Increasing UV-B exposition inhibited young maca leaves expansion without visible stress symptoms, but with a marked induction of serrated leaves formation. It has been demonstrated the sensitivity of leaf shape to environmental conditions involving several molecular pathways [1]. For instance, UV-B can induce changes on plant morphology via specific signaling and/or generic-mediated pathways [28]. Interestingly, a recent genome study of maca grown at 4200 m of altitude in Yunnan (China) identified several genes involved in leaf morphogenesis and UV-B stress response [40]. However, the effectiveness of maca serrated leaf in conferring protection against UV radiation requires further investigation. Our results also showed that prolonged UV-B exposure reduced Φ_PSII_ and *F*_*v*_*/F*_*m*_ values (Fig. 4), indicating an inhibition of the photochemistry activity due to detrimental effects on the functioning of PSII. Moreover, a reduction in photosynthetic CO_2_ uptake was observed without significant changes in stomatal conductance and increased intercellular CO_2_ concentration (Fig. 5), suggesting that the decline in photosynthesis may be due to non-stomatal factors associated with acute UV-B-induced ROS deleterious effects on chlorophylls, PSII components and Rubisco activity, which are also indicators of senescence observed in the well-expanded leaves [15, 32]. Consequently, low photosynthetic carbon fixation affects the balance between carbon assimilation, storage and growth in plants [31], a phenomenon observed in maca under prolonged acute UV-B irradiation (Fig. 1–5). Thus, the reduction of underground biomass could be attributed to the trade-off between carbon allocation to plant growth and plant protection in response to UV-B irradiation. However, we do not discard the possibility that maca root growth inhibition may be induced by UV-B increased antioxidants, such as flavonoids, which can modulate phytohormones affecting root development [20]. Interestingly, a transient increase of dark respiration was observed with UV-B exposition from 1 to 10 days of treatment (Fig. 6), which may be related to a higher energy demand to counteract UV-B damages and to allow carbon conversion to secondary compounds associated with UV-B-absorbing compounds [41].

Since plants need to cope with irregular environmental changes, such as occasional UV-B exposure under field conditions, we evaluated the capacity of maca plants to recover after UV-B irradiation. At first, control plants showed an increase of underground biomass and a reduction of epigeal organ (Fig. 1) constituted of both new and age-induced leaf senescence (Additional file 1: Figure S1). This phenotype suggests that maca, at this stage of development, apparently remobilized and relocated nutrients from old leaves to new leaf development and storage organs in a coordinated manner. Interestingly, recovery of 1 d UV-B plants increased biomass with respect to the control mainly due to their underground organs (Fig. 1); moreover, they maintained similar photosynthetic efficiency like the control but promoting the carbohydrate allocation in the reserve organs (Figs. 2–5). This strategy is usually adopted by perennial plants in which roots are important nutrients sinks that later are used during the recovery after stress [29]. Taking into consideration that maca is an herbaceous perennial plant, let us to speculate that non-acclimated maca may ameliorate the sudden acute UV-B irradiation through source to sink allocation. A different response was observed in recovered plants of 3 d UV-B, which highly decreased their biomass (Fig. 1) with an accelerated leaf senescence process in UV-B exposed and well-expanded leaves (Additional file 1: Figure S1). This senescence acceleration may be related to PCD in response to repeated UV-B exposure associated with the accumulation of ROS. Thus, maca plants seem to avoid ROS propagation by the induction of PCD in affected organs (old leaves) allowing nutrients remobilization to the development of new leaves, as observed in other highland species [37]. Interestingly, here maca did not allocate its resources in the underground organs (Figs. 2 and 3), like the control and recovered 1 d UV-B plants, probably due to a convergence between UV-B-specific and generic signaling pathways. We proposed that whereas in old leaves an increase ROS production occurred, new leaves may activate UV-B adaptation mechanisms by a systemic defense response prior to emergence [4], which at the same time may modulate the root development [20]. Interestingly, 1 and 3 d-treated plants showed a full recovery of PSII photochemistry (Fig. 4), suggesting that plants activated UV-B-induced compounds, such as flavonoids, antioxidants, etc. [16, 36], which protect the photosynthetic apparatus from permanent damage. Subsequent recovery in 7 to 10 d UV-B plants showed a negative correlation between increased UV-B and recovery with marked production of new biomass (Additional file 1: Figure S1), suggesting a possible acclimation of maca plants to prolonged UV-B irradiation. Moreover, a pronounced leaf morphogenesis was observed by the induction of serrated leaves, phenomenon observed also under natural environment [40] and probably linked to UV-B-specific signaling [14]. Conversely, this morphogenic strategy in maca did not affect the efficiency of the photosynthesis and the resources were balanced between above and underground organs (Figs. 2–5), reinforcing the hypothesis of acclimation of these plants. Finally, recovery in 14 d UV-B plants presented similar biomass distribution as 3 d UV-B after recovery (Fig. 1); however, epigeal organs were only constituted by new leaves (Additional file 1: Figure S1). Moreover, even though these plants maintained high efficiency in photosynthesis (Figs. 4 and 5), their carbohydrate concentration was dramatically reduced (Figs. 2 and 3). This fact could be mainly related to the lethal UV-B damage not only in old but also in new leaves just after treatment, leading to a reduction of new leaf number and a delay of their expansion in comparison with that of the 7 and 10 d UV-B plants after recovery (Additional file 1: Figure S1). Here a second threshold may happen for maca plants in response to repeated acute UV-B irradiation, where the plant development is compromised by the reduced energetic status. MFA enabled the set of observations based on biometric and physiological data to be analyzed within the same framework, thus giving an integrated picture of the observations and the relationships among the variables recorded. After treatments, the joint analysis of the variables performed confirmed that there was a marked similarity between the treatments with exception of the control, 1 and 14 days of UV-B treatment. In contrast, the analysis performed after recovery led to the separation of the plants subjected to the highest exposure time (10 and 14 days of UV-B treatment) with respect to the performance obtained.

## Conclusions

We have found that maca is able to modulate different defense mechanisms in response to repeated acute UV-B irradiation by the activation of specific and/or generic pathways. Similar modulation was also observed in our previous study in quinoa plants, another Andean crop [11]. However, quinoa was more sensitive to acute UV-B irradiation (6.08 kJ m^− 2^ d^− 1^) than maca plants, which showed a better capacity to recover their photosynthetic performance after strong stress. Thus, UV-B stress response was mainly controlled by a coordinated source-sink nutrient allocation, while acclimation process may require an UV-B-specific systemic defense response reflected on the phenotypic plasticity of maca plants under controlled conditions. All these modulations may be associated to maca adaptation in response to harsh environments within its short geological period in the Andes. Further studies are ongoing to evaluate these results under natural environment.

## Methods

### Plant material and growth conditions

Seeds of yellow maca were obtained from a local farmer in Junín (Peru). Seeds were sterilized and sown on MS medium as described [11]. After 14 days, seedlings were transferred in plastic pots containing commercial soil and grown at 12 h photoperiod, temperature 22 ± 1 °C, relative humidity 50% and Photosynthetic Photon Flux Density (PPFD) of 100 μmol quanta m^− 2^ s^− 1^. Plants received distilled water three times a week.

### UV-B irradiation treatment

One month-old plants were subjected to UV-B treatment as previously described [11]. Briefly, UV-B radiation was generated using three UV-B lamps (Philips TL 20 W/01RS UV-B Narrowband, Koninklijke Philips Electronics, Eindhoven, The Netherlands) with a narrow waveband between 305 and 315 nm and peak emission at 311 nm. The spectra of the UV-B lamps were measured using a JAZ EL200-XR1 spectroradiometer (Ocean Optics, Dunedin, FL, USA). The dose of the UV-B radiation was 6.08 kJ m^− 2^ d^− 1^ and plants were daily irradiated in the middle of the light period for 60 min following a time course from 1 to 14 days. Treated plants received the same PPFD of 100 μmol quanta m^− 2^ s^− 1^ as control ones, even during UV-B treatment. For recovery test, a set of UV-B treated and untreated plants was transferred to control conditions for two weeks.

### Biometric analysis

Two different sets of plants were used for testing biometric traits: just after treatment or after 2-weeks of recovery. UV-B treated and untreated plants were separated into epigeal, hypocotyl and root, and then weighed (fresh weight, FW). Hypogeal tissues were previously washed with water to remove the soil. After FW registration, all vegetal materials were immediately frozen in liquid nitrogen and stored at − 80 °C for further biochemical analyses.

### Total soluble sugars quantification

Epigeal, hypocotyl and root samples were ground to a powder in liquid nitrogen, and then extracted and assayed for total soluble sugar (TSS) content through coupled enzymatic assay methods as described [12]. The quantity of TSS was expressed as μmol hexose equivalents g^− 1^ FW. In addition, TSS amount was expressed per FW of corresponding organ (μmol g^− 1^ FW of corresponding organ), since some differences were detected among total FW biomass of each organ (whole leaves, hypocotyl and whole roots) of single plant.

### Starch analysis

Starch content was only evaluated in the hypocotyls which are the main reserve organs. Starch was extracted and analyzed as described [24] with minor modifications. Hypocotyl samples were ground in a mortar, resuspended in 5 mL of 10 mM KOH and boiled for 1 min. 50 μL of 1 N HCl was then added to each sample. The starch standard solution was prepared using 10 mg of potato soluble starch dissolved in 10 mL distilled water and boiled 1 min. Standards (from 0 to 100 μL) were adjusted to 150 μL with neutralized solution (10 mM KOH:1 N HCl, 100:1). 1 mL of fresh iodine solution (1.3% K_2_ and 3% KI, dissolved in distilled water) was added into aliquots of samples (150 μL) and standards. The absorbance was immediately read at 595 nm and the starch concentration was expressed as mg starch g^− 1^ FW and mg starch g^− 1^ FW of corresponding organ.

### Chlorophyll *a* fluorescence and leaf gas exchange

Chlorophyll *a* fluorescence and leaf gas exchanges were simultaneously measured using an open-air-circuit type portable measurement system (Li-6400, Li-Cor Inc., NE, USA) equipped with an integrated fluorescence chamber head (Li-6400-40 leaf chamber fluorometer, Li-Cor Inc.) as previously described [21]. Measurements were carried out on fully expanded and exposed leaves in both untreated and 1, 3, 7, 10 and 14 days UV-B treated plants. The same measurements were performed also after 2 weeks of recovery following the UV-B treatment on fully expanded leaves. Instantaneous measurements of steady state photosynthetic CO_2_ assimilation rate (*A*), stomatal conductance (*g*_*s*_), intercellular CO_2_ concentration (*C*_*i*_), transpiration rate (*E*) and actual photon yield of PSII photochemistry (Φ_PSII_) were determined at CO_2_ concentration of 400 μmol mol^− 1^, relative humidity ranging between 45 and 55%, leaf temperature of 22 °C and light intensity of 100 μmol quanta m^− 2^ s^− 1^, as described [5]. All the gas exchange and fluorescence data were recorded at steady state, allowing the leaves to adapt inside the chamber to the above conditions for about 5 min for adjustment and stabilization of the parameters [30]. The value of Φ_PSII_ was determined as Φ_PSII_ = (*F*_*m*_*′*–*F′*)/*F*_*m*_*′* [7], where *F′* is the fluorescence yield emitted by the leaves under actinic light exposition, whereas *F*_*m*_*′* is the maximum fluorescence yield emitted after superimposing a saturating light flash during actinic illumination. The maximum PSII photochemical efficiency (*F*_*v*_/*F*_*m*_) and the dark respiration (*R*_*d*_) were determined on leaves after at least 30 min of dark acclimation [30]. The value of *F*_*v*_/*F*_*m*_ was determined as *F*_*v*_/*F*_*m*_ = (*F*_*m*_ – *F*_*0*_)/*F*_*m*_, where *F*_*m*_ and *F*_*0*_ represent the maximum and the minimum fluorescence yield emitted by the leaves in the dark-adapted state, respectively.

### Statistical analysis

Pots were arranged in a randomized complete-block design. Three biological replicates were considered for each time point. Following Bartlett’s test for homogeneity of variance, data were subjected to one-way analysis of variance (ANOVA). Tukey’s HSD tests were used to identify statistically different means in the other response variables, using the *multcomp* package [10]. Probability levels lower than 0.05 were categorized as significant.

To identify relationships among the different UV-B treated plants observed during the time course experiment, based on biometric and overall physiological data obtained before and after recovery, multiple factorial analyses (MFA) were carried out [25], implemented in the R packages *FactoMineR* [17]. Basically, each exposure time had four partial points corresponding to the trait classes (biometric, starch, TSS, and gas exchange). Trait classes that significantly contributed to MFA dimensions were used to explain differences among exposure times (*α* = 0.05). The length and the direction of the vectors were directly correlated to their significance within each exposure time. Finally, a hierarchical clustering on principal components (HCPC) was performed to confirm the product groups observed graphically. All computations were performed using the R 3.4.3 language and environment [27], and the R data visualization package *ggplot2* [38] was used.

## Additional files

Additional file 1: **Figure S1.** Representative phenotypes of UV-B-treated and untreated maca before and after recovery. (PDF 319 kb)

Additional file 2: **Figure S2.** UV-B induced reactive oxygen species (ROS) generation in maca. (PDF 131 kb)

## Abbreviations

*A*: CO_2_ assimilation rate
*C*_*i*_: Intercellular CO_2_ concentration
DOT: Days of treatment
*E*: Transpiration rate
*F*_*v*_*/F*_*m*_: Maximum efficiency of PSII photochemistry
FW: Fresh weight
*g*_*s*_: Stomatal conductance
HCPC: Hierarchical clustering on principal components
MFA: Multiple factor analysis
PCD: Programme cell death
PPFD: Photosynthetic photon flux density
PSII: Photosystem II
*R*_*d*_: Dark respiration
ROS: Reactive oxygen species
TSS: Total soluble sugars
UV: Ultraviolet
UVR8: UV-B resistance locus 8
Φ_PSII_: Actual efficiency of PSII photochemistry
