# Supplementary material for: Physiological responses of Lepidium meyenii plants to ultraviolet-B radiation challenge
Source: BMC Plant Biol. 2019 May 7;19:186. doi: 10.1186/s12870-019-1755-5 (PMC6505108; doi:10.1186/s12870-019-1755-5)
Supplement: Supplementary file 1 — Figure S1. Representative phenotypes of UV-B-treated and untreated maca before and after recovery. (PDF 319 kb) [file 12870_2019_1755_MOESM1_ESM.pdf]

Physiological responses of *Lepidium meyenii* plants to ultraviolet-B radiation challenge

Huarancca Reyes T[1*], Scartazza A[2], Pompeiano A[3], Guglielminetti L[1,4]

[1]Department of Agriculture, Food and Environment, University of Pisa, Via del Borghetto 80, 56124 Pisa, Italy

[2]Institute of Research on Terrestrial Ecosystems, National Research Council, Via Salaria km 29,300, 00015 Monterotondo Scalo (RM), Italy

[3]Center for Translational Medicine (CTM), International Clinical Research Center (ICRC), St. Anne's University Hospital, 62500, Brno, Czech Republic

[4]Interdepartmental Research Center "Nutraceuticals and Food for Health", University of Pisa, Via del Borghetto 80, 56124 Pisa, Italy.

*Corresponding author: thais.huarancca@agr.unipi.it

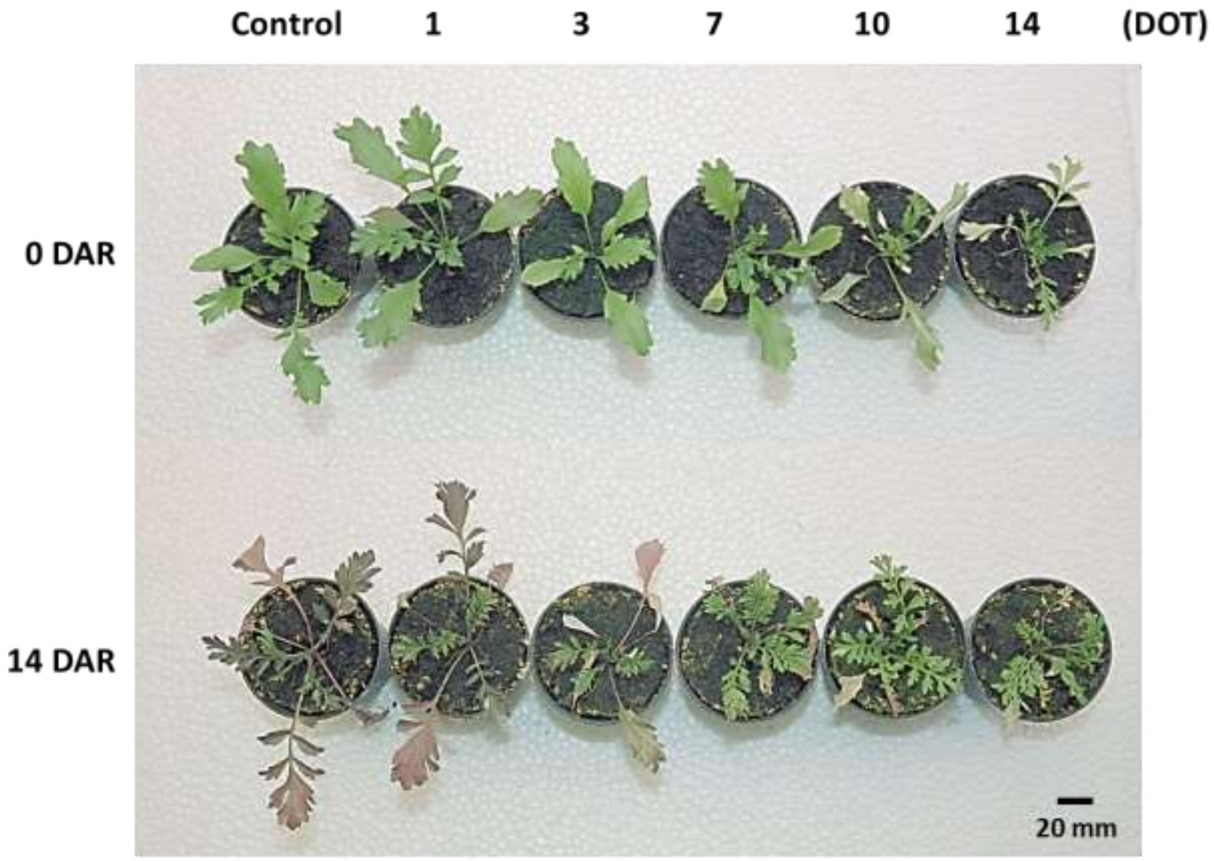

Fig. S1 Representative phenotypes of UV-B-treated and untreated maca before and after recovery. Treated plants were exposed to 100 µmol m^{-2} s^{-1} and 6.08 kJ m^{-2} d^{-1} UV-B. UV-B was daily applied following a time course from 1 to 14 days. Control plants did not receive UV-B radiation. For recovery test, UV-B treated plants were transferred to control conditions for two weeks. DOT, days of UV-B treatment. DAR, days after recovery.
